# The Hemodynamic Effect of Enhanced External Counterpulsation Treatment on Atherosclerotic Plaque in the Carotid Artery: A Framework of Patient-Specific Computational Fluid Dynamics Analysis

**DOI:** 10.1155/2020/5903790

**Published:** 2020-04-30

**Authors:** Jianhang Du, Guangyao Wu, Bokai Wu, Chang Liu, Zhouming Mai, Yumeng Liu, Yawei Wang, Pandeng Zhang, Guifu Wu, Jia Liu

**Affiliations:** ^1^Department of Cardiology, The Eighth Affiliated Hospital of Sun Yat-sen University, Shenzhen 518033, China; ^2^Guangdong Innovative Engineering and Technology Research Center for Assisted Circulation (Sun Yat-sen University), Shenzhen 518033, China; ^3^NHC Key Laboratory of Assisted Circulation (Sun Yat-sen University), Guangzhou 510080, China; ^4^Department of Radiology, Shenzhen University General Hospital, Shenzhen 518055, China; ^5^Laboratory for Engineering and Scientific Computing, Shenzhen Institutes of Advanced Technology, Chinese Academy of Sciences, Shenzhen 518055, China; ^6^School of Biological Science and Medical Engineering, Beihang University, Beijing 100083, China; ^7^Shenzhen Key Laboratory for Exascale Engineering and Scientific Computing, Shenzhen, China

## Abstract

Long-term enhanced external counterpulsation (EECP) therapy has been recommended for antiatherogenesis in recent clinical observations and trials. However, the precise mechanism underlying the benefits has not been fully clarified. To quantify the effect of EECP intervention on arterial hemodynamic environment, a framework of numerical assessment was introduced using a parallel computing algorithm. A 3D endothelial surface of the carotid artery with mild atherosclerotic plaque was constructed from images of magnetic resonance angiography (MRA). Physiologic boundary conditions were derived from images of the ultrasound flow velocity spectrum measured at the common carotid artery and before and during EECP intervention. Hemodynamic factors relating to wall shear stress (WSS) and its spatial and temporal fluctuations were calculated and analyzed, which included AWSS, OSI, and AWSSG. Measuring and computational results showed that diastole blood pressure, perfusion, and WSS level in carotid bifurcation were significantly increased during EECP intervention. Mean AWSS level throughout the model increased by 16.9%, while OSI level did not show a significant change during EECP. We thus suggested that long-term EECP treatment might inhibit the initiation and development of atherosclerotic plaque via improving the hemodynamic environment in the carotid artery. Meanwhile, EECP performance induced a 19.6% increase in AWSSG level, and whether it would influence the endothelial functions may need a further study. Moreover, the numerical method proposed in this study was expected to be useful for the instant assessment of clinical application of EECP .

## 1. Introduction

As a kind of noninvasive and atraumatic assisted circulation procedure, enhanced external counterpulsation (EECP) has exhibited itself to be an effective, safe, and economical therapy in clinics for the management of ischemic cardiovascular and cerebrovascular diseases in the recent decades [1–5] and has been thought providing a better choice for patients with chronic stable angina who failed to respond to standard revascularization procedures and aggressive pharmacotherapy [6].

**Figure 1:**
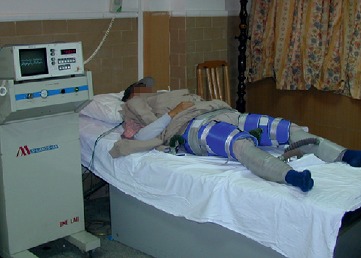
EECP treatment in clinics and animal experiment [6]. The technique involves the using of a set of cuffs that are wrapped around the lower parts of the body and connected to an air compressor with tubes.

The treatment of EECP (see Figure 1) involves the use of an EECP device to inflate and deflate a series of compressive cuffs wrapped around the patient's calves, lower thighs, and upper thighs. As a result, the enhanced flow perfusion is achieved from the device's propelling blood from veins of the lower body to arteries of the upper body and increases the blood supply for the important organs and the brain [7].

Long-term EECP intervention has been demonstrated in recent studies to be able to improve the endothelial functions and in turn may inhibit the generation and development of atherosclerosis lesion [8–11]. The hemodynamic effects, especially the wall shear stress variations, induced by EECP have been thought contributing the most important part of its benefits. Michaels et al. [12] confirmed that EECP treatment could significantly increase coronary artery flow determined by both Doppler and angiographic techniques. Braith et al. [10] suggested that EECP had a beneficial effect on peripheral artery flow-mediated dilation and endothelial-derived vasoactive agents. Our previous study [8] experimentally confirmed that EECP inhibits intimal hyperplasia and atherogenesis by modifying biomechanical stress-responsive gene expression. However, the actual influence of EECP intervention on wall shear stress (WSS) and its spatial and temporary fluctuations remained elusive.

It has been widely accepted that biomechanical stresses of large and medium arteries play an important role in maintaining the functions of endothelium and vascular remodeling progression [13]. Low and/or oscillating WSS has been commonly believed to be correlated positively with initiation and development of atherosclerosis [14–16]. Several hemodynamic factors have been proposed by different research groups to represent the biomechanical indicators connected to arterial functions, such as average wall shear stress (AWSS), oscillatory shear index (OSI), particle resident time (PRT), and wall shear stress gradient (WSSG).

This paper was aimed to conduct a pilot study on how the EECP treatment affects the hemodynamic environment and the important factors in carotid arterial bifurcation where the atherosclerotic lesion localizes characteristically. A numerical method-combined finite element method with *in vivo* medical imaging measurement was introduced to assess the local hemodynamic factors during EECP intervention.

## 2. Medical Image Acquisition and Processing

A 55-year-old coronary heart disease patient with mild carotid atheromatous plaque diagnosed (severity of stenosis was less than 20%) was enrolled to the measurement. The subject underwent the clinical protocol for carotid plaque MRI on a 3T MRI (General Electric Company, Discovery MR750). A two-element bilateral 8-channel carotid surface coil (Wk401 Jiangyin Wankang Medical Technology Co., Ltd.) was used for image acquisition.

A three-dimensional phase-contrast magnetic resonance angiography (3D Phase Contrast) sequence was performed: repetition time/echo time (TR/TE) 15.0/3.7 ms; flip angle 8°, field of view 32 cm × 32 cm, slice thickness 1.8 mm, and matrix 384 × 256. A High Resolution Three-Dimensional CUBE (computer use by engineers) T1 Weighted Imaging(HR 3D CUBE T1WI) sequence was performed as follows: repetition time/echo time (TR/TE) 575/15 ms; echo chain length (ETL) 24, slice thickness 0.8 mm, field of view 28  cm × 28 cm, and matrix 256 × 256).

## 3. EECP Intervention Protocol and Color Doppler Ultrasound Measurement

A short-term EECP intervention was performed using Pushikang P-ECP/TM Oxygen Saturation Monitoring Enhanced External Counterpulsation Instrument (made in Chongqing, China). The subject received a single, 45-minute session EECP treatment with the working pressure set to 0.033 MPa.

**Figure 2:**
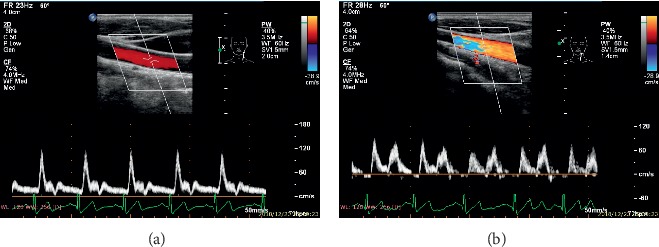
Blood flow velocity and spectrum measurement based on Color Doppler Ultrasound. (a) pre-EECP intervention. (b) During EECP intervention. Note that EECP significantly changed the blood flow pattern and increased the blood flow level in diastole.

**Figure 3:**
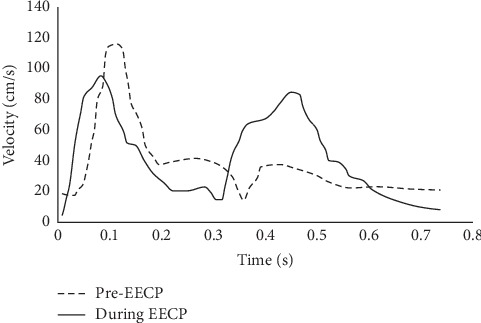
Blood velocity waveforms at CCA in a cardiac cycle before and during EECP, which were extracted from the images of ultrasound flow velocity spectrum.

**Figure 4:**
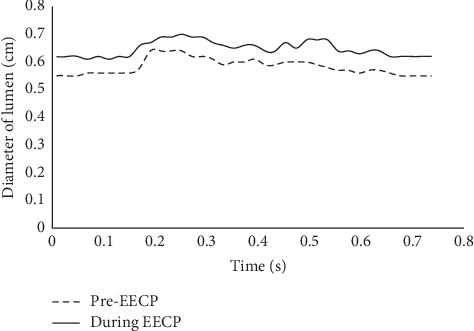
Diameter of the lumen at CCA in a cardiac cycle before and during EECP, which were extracted from the images of ultrasound carotid artery.

The blood velocity measurements of before EECP (rest state) and during EECP (15-25min after EECP initiated) were performed based on a Color Doppler Ultrasound System (Philip EPIQ7). (see Figure 2). The left common carotid arteries (CCA) were examined with 1.5 cm proximal to the bifurcation of the vessels. The blood velocity waveforms (see Figure 3) in cardiac cycles and before and during EECP intervention were extracted from the images of ultrasound flow velocity spectrum. Meanwhile, the diameter changes of the lumen section (see Figure 4) in cardiac cycles were extracted from images of ultrasound. The blood flow rate in cardiac cycle and perfusion in CCA could be calculated based on velocity waveforms and diameter changes.

## 4. 3D Reconstruction for the Endothelial Surface of the Carotid Artery

**Figure 5:**
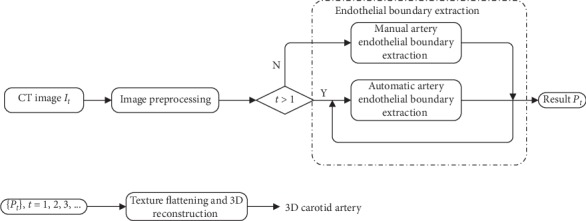
The pipeline of our algorithm.

We propose a method to virtually reconstruct the endothelial surface of the carotid artery so as to visualize the carotid atheromatous plaque in 3D. The pipeline of our work is shown in Figure 5, where *I*_*t*_ denotes the input MR image with index *t*, *t* = 1, 2, 3,…; *P*_*t*_ represents the artery endothelial boundary extracted from *I*_*t*_. This pipeline consists of three main steps: image preprocessing, endothelial boundary extraction, and 3D reconstruction.

## 5. Image Preprocessing

**Figure 6:**
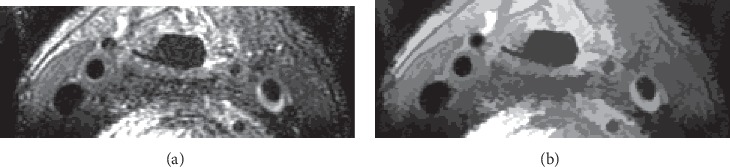
(a) The original MR image. (b) The result of morphological reconstruction.

There exists serious inherent noise in the MR image, as shown in Figure 6(a). The noise has a detrimental influence on the accuracy of the artery extraction and 3D reconstruction. In order to reduce the noise without affecting the shape of the carotid artery, we make use of the morphological technique called open-by-reconstruction and close-by-reconstruction [17]. The processed result is illustrated in Figure 6(b). It can be seen that most of the clutters in Figure 6(a) have been removed, and the shape of the carotid artery is not influenced.

### 5.1. Endothelial Boundary Extraction

**Figure 7:**
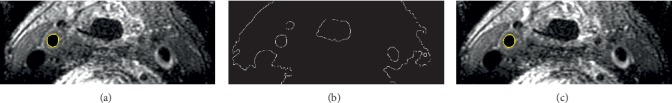
Automatic endothelial boundary extraction. (a) The result *P*_*t*−1_. (b) The edge map of the binarized *I*_*t*_. (c) The result *P*_*t*_.

The extraction of the endothelial boundary of the carotid artery in each MRA image is implemented based on the result of image preprocessing. For *t* = 1, we manually extract the boundary of the carotid artery in *I*_1_, so as to initialize the subsequent automatic extraction. If *t* > 1, given the extraction result *P*_*t*−1_ (the yellow curve in Figure 7(a)),we first binarize the *I*_*t*_ preprocessed and then obtain the edge map *E* from this binary image (Figure 7(b)). Because of the great similarity between *I*_*t*_ and *I*_*t*−1_, we eventually optimize a closed curve *c* = [*x*, *y*], which satisfies the following equation with calculus of variations using *P*_*t*−1_ as the initial value.

(1) $$\begin{array}{c} \min\oint \alpha \left\|{\frac{\text{d}c\left(s\right)}{\text{d}s}}^{2}\right\|+\beta \left\|{\frac{{\text{d}}^{2}c\left(s\right)}{\text{d}s^{2}}}^{2}\right\|+\gamma \left(1-E\left(x\left(s\right),y\left(s\right)\right)\right)\text{d}s, \end{array}$$

where *s* is the arc parameter of *c*, and *α*, *β*, as well as *γ* are the three scalars to balance the three terms in equation (1). The optimized *c* is the result *P*_*t*_ of *I*_*t*_, as shown by the yellow curve in Figure 7(c). Equation (1) can be calculated by the method proposed in [18].

### 5.2. Texture Flattening and 3D Reconstruction

**Figure 8:**
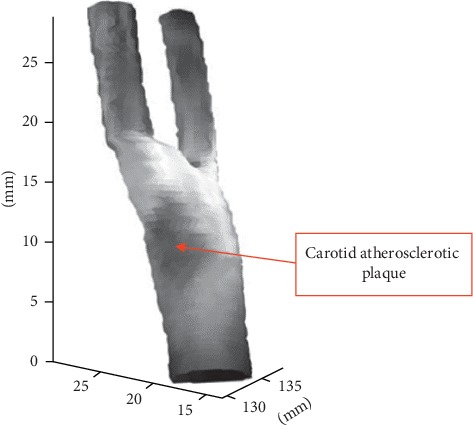
The reconstruction of the carotid artery.

The carotid artery can be approximately considered as a surface of revolution (SOR). Providing that the endothelial boundary of the carotid artery in each MRI slice has been extracted, the algorithm proposed in [19] can be adopted to flatten the texture of the endothelial surface of the carotid artery and then generate a 3D texture reconstruction for the endothelial surface. The carotid artery will bifurcate at its end, so we can generate the 3D texture reconstruction for each bifurcation by the same way and finally combine all reconstructions together. The final reconstruction of the carotid artery is demonstrated in Figure 8. In this figure, the dark and concave region is the carotid atherosclerotic plaque.

## 6. The CFD Method and the Boundary Conditions

### 6.1. Geometry and Boundary Conditions

**Figure 9:**
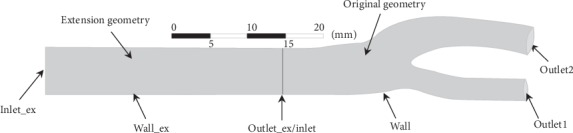
Geometry and boundaries of the carotid artery.

To simplify simulations, the elasticity of vessel wall is not considered in the present study (i.e., computational domain was fixed). Original geometry consists of four boundaries: inlet, outlet, and wall. An artificial extension geometry, with a length of 5 times the averaged radius of the inlet is added outward along the normal direction of the inlet boundary to acquire a fully developed velocity waveform [15], as depicted in Figure 9. Inflow velocity (*V*_in_) measured by the carotid Doppler (see Figure 3) is specified at the inlet_ex. Opening condition is set at the outlet, and wall is assumed to be no-slip.

### 6.2. Mesh Generation

**Figure 10:**
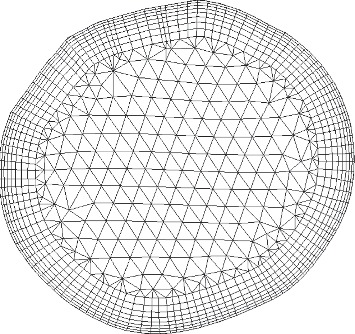
Schematic mesh at the inlet.

Most part of the geometry is meshed with tetrahedral cells by employing the commercial software ANSYS ICEM (ANSYS, Inc., USA). To capture the flow behavior where high velocity gradient exists, inflation layers are created near the wall [16] (as shown in Figure 10). To optimize the mesh size, a specific mesh-independent study is carried out for reliable results, while keeping computational loads as low as possible. As indicated in Table 1, change in AWSS is around 3% with refinement from Mesh 1 to Mesh 3, while less than 1% from Mesh 3 to Mesh 4 for both states with and without EECP. Mesh used in this study is of quantity 720085 (i.e., Mesh 3) and with a quality of ∼0.4 (measured by its orthogonality and warpage), which is at an acceptable level.

### 6.3. Rheology and Governing Equations

The blood fluid used in the present study is assumed to be impressible and isoviscous (i.e., Newtonian type). Therefore, the governing transport equations for this study are continuity and momentum equations which can be written in their general forms [20], as follows:

(2) $$\begin{array}{c} \text{continuity}:\;\nabla \cdot v=0, \end{array}$$

(3) $$\begin{array}{c} \text{momentum}:\;\frac{\partial v}{\partial t}+\left(v\cdot \nabla \right)v=-\frac{1}{\rho }\nabla p+\frac{\mu }{\rho }\nabla^{2}v+f, \end{array}$$

where *ν* is the velocity vector, *p* is the pressure, *ρ* is the fluid density, and *f* is the external force (assumed to be 0 here).

### 6.4. The solver

The governing equations are discretized by CFX code based on a finite-volume method. To meet the requirement on both robustness and accuracy, a so-called “High Resolution Advection Scheme” is implemented in this study [21]. Numerical solutions are acquired while root mean square (RMS) of both mass and momentum residuals are below 10^−5^. In fact, however, at most time steps, even lower RMS residual values are generally reached. We solve the unknowns with this configuration for four cardiac cycles. Results of the last cardiac cycle are presented in the following section.

## 7. Results

Several important hemodynamic factors such as AWSS, OSI, RRT, and WSSG were calculated in this paper, which were introduced by different research groups to represent the WSS level in a cardiac cycle and its spatial and temporary fluctuation. These factors are defined as follows [22, 23]:

(4) $$\begin{array}{c} \text{AWSS}=\frac{1}{T}\int_{0}^{T}\left|{\overset{⟶}{\tau }}_{w}\right|\text{d}t, \end{array}$$

(5) $$\begin{array}{c} \text{WSSG}=\sqrt{{\left(\left|\frac{\partial {\overset{⟶}{\tau }}_{w}}{\partial x}\right|\right)}^{2}+{\left(\left|\frac{\partial {\overset{⟶}{\tau }}_{w}}{\partial y}\right|\right)}^{2}+{\left(\left|\frac{\partial {\overset{⟶}{\tau }}_{w}}{\partial z}\right|\right)}^{2}}, \end{array}$$

(6) $$\begin{array}{c} \text{AWSSG}=\frac{1}{T}\int_{0}^{T}\text{WSSG d}t, \end{array}$$

(7) $$\begin{array}{c} \text{OSI}=\frac{1}{2}\left(1-\frac{\left|\int_{0}^{T}{\overset{⟶}{\tau }}_{w}\text{d}t\right|}{\int_{0}^{T}\left|{\overset{⟶}{\tau }}_{w}\right|\text{d}t}\right), \end{array}$$

(8) $$\begin{array}{c} \text{RRT}=\frac{1}{\left(1-2\text{OSI}\right)\times \text{AWSS}}, \end{array}$$

where $\left|{\overset{⟶}{\tau }}_{w}\right|$ is the magnitude of the instantaneous WSS vector ${\overset{⟶}{\tau }}_{w}$ and *T* is the cardiac cycle.

**Figure 11:**
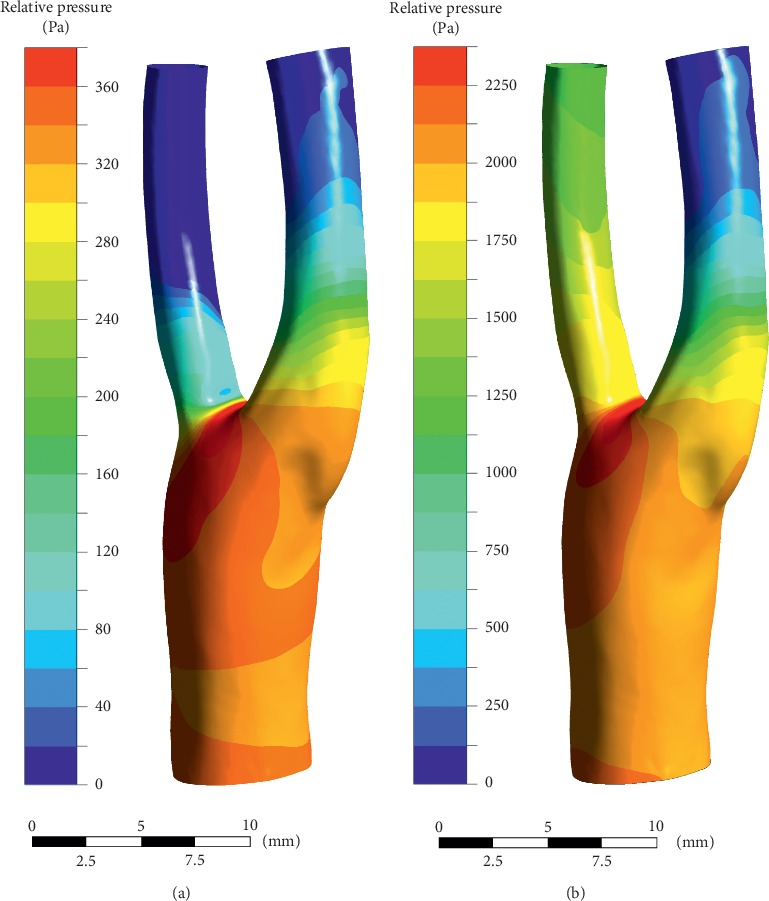
Relative blood pressure distributions in diastole. (a) Pre-EECP, *t* = 0.54*T*. (b) During EECP, *t* = 0.58*T*. Note that EECP intervention significantly increased the blood pressure in diastole.

**Figure 12:**
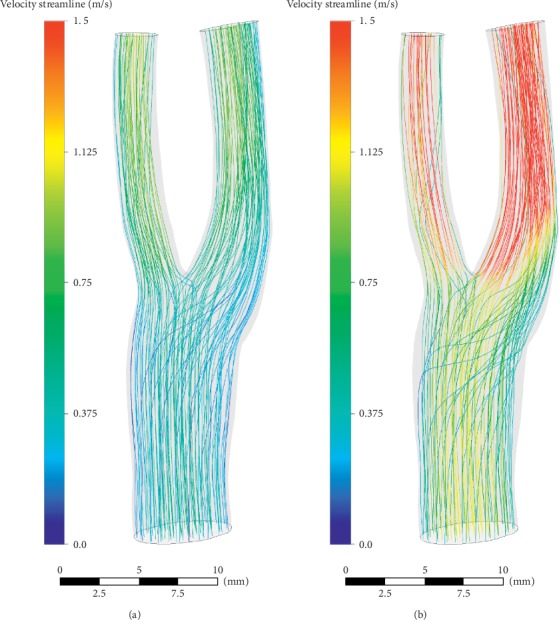
Blood velocity distributions in diastole. (a) Pre-EECP, *t* = 0.54*T*. (b) During EECP, *t* = 0.58*T*. Note that EECP intervention significantly increased the blood velocity in diastole.

**Figure 13:**
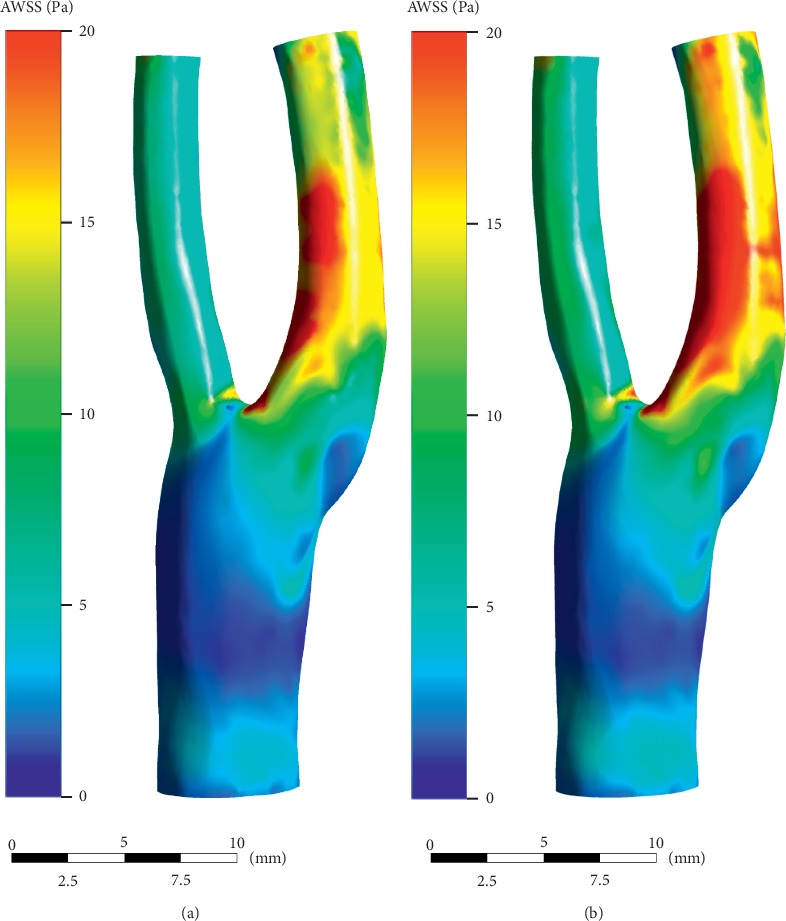
AWSS distributions over the cardiac cycle. (a) Pre-EECP. (b) During EECP. Note that high level AWSS occurred at the internal carotid artery (ICA), whereas low level AWSS occurred at bulb. EECP intervention significantly increased the AWSS level at sinus of the bifurcation.

**Figure 14:**
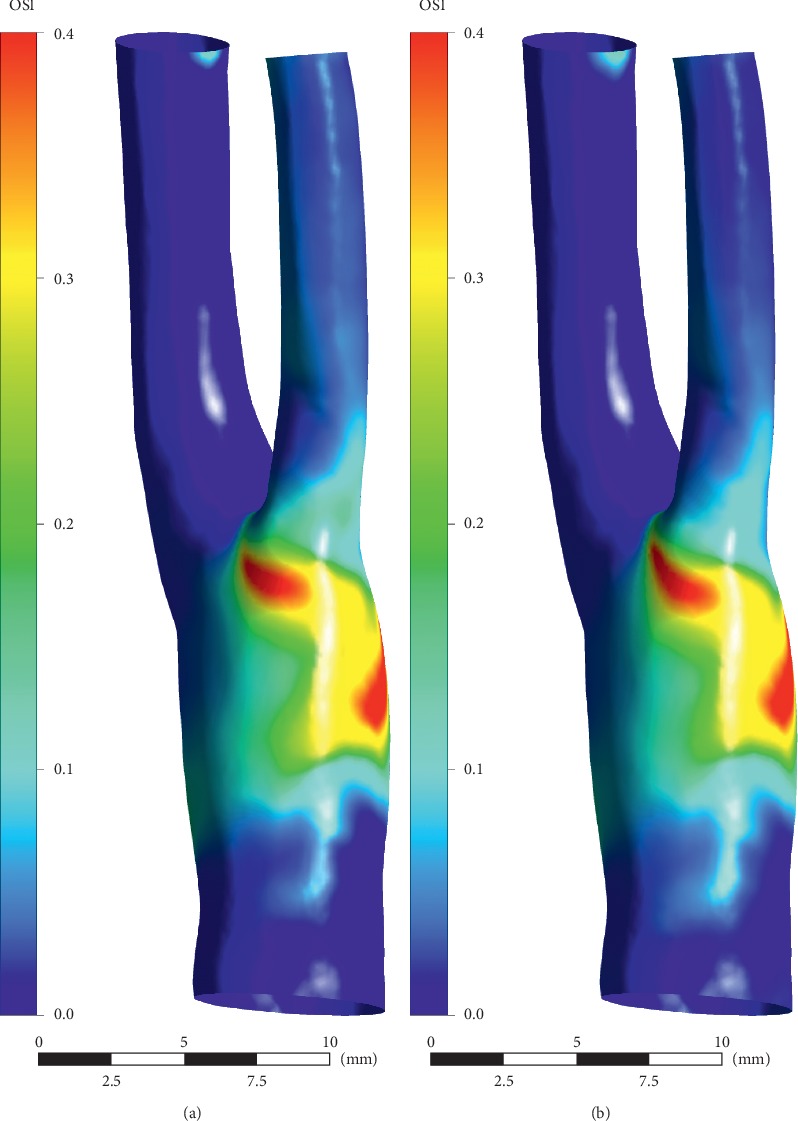
OSI distributions over the cardiac cycle. (a) Pre-EECP. (b) During EECP. Note that high level OSI occurred at bulb, and EECP intervention didn't induce significant change of OSI level.

**Figure 15:**
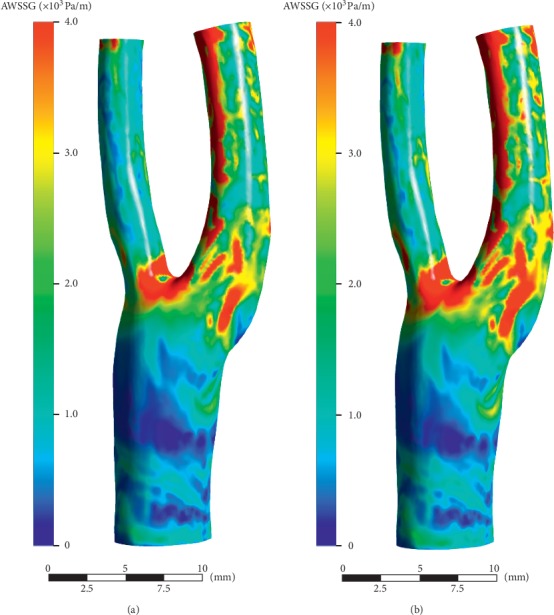
AWSSG distributions over the cardiac cycle. (a) Pre-EECP. (b) During EECP. Note that high level AWSS mainly occurred at bifurcation site and ICA. Meanwhile, EECP intervention slightly increased the AWSSG level.

The numerical results are shown in Figures 11–15. Focus of previous studies were mainly put on how EECP affected the blood flow and pressure in diastole [1, 12]. In the current paper, the velocity and pressure at time points of *t* = 0.54*T* and *t* = 0.58*T* were chosen to represent the blood velocity and pressure in diastole before and during EECP intervention respectively, considering that peak blood velocity in diastole occurred at these time points based on Figure 3.

## 8. Discussions

The computational results are summarized in Table 2, which include AWSS, OSI, and AWSSG over the cardiac cycle and before and during EECP intervention. All calculations and statistics were performed based on the whole model.

The results showed that EECP performance significantly increased the blood velocity in diastole, as well as the blood pressure. Peak relative pressure during EECP increased about 2260 Pa comparing to pre-EECP state. The elevation of diastolic pressure was thought playing a key role for the increasement of perfusion [24]. A calculation based on Figures 3 and 4 showed that EECP performance induced a 12.6% elevation of the perfusion over a cardiac cycle in CCA.

WSS has been widely recognized to be an important hemodynamic factor affecting the initiation and development of atherosclerotic plaque. It is now well accepted that low and oscillating WSS correlate positively with atherosclerosis progression [25]. Our calculating results showed that EECP performance significantly increased the WSS level in carotid bifurcation and especially in bulb and ICA, and the mean AWSS level throughout the model increased by 16.9% (7.90 Pa versus 6.76 Pa).

As a factor proposed to represent the temporary fluctuation of WSS, OSI has been found to be associated with early atherosclerosis in some studies, and region of high OSI coincides with a high probability of occurrence of early atherosclerosis lesions [26, 27]. Our results showed that EECP performance didn't induce a significant change in OSI level in the carotid artery, although this kind of intervention greatly changed the blood flow pattern.

As a factor proposed to represent the spatial fluctuation of WSS, WSSG has been suggested in some studies that might correlate with intima-medial thickness and endothelial dysfunction [27, 28]. Our current study showed that EECP performance induced a significant increase in WSSG level in the carotid artery. The mean and peak AWSSG level throughout the model, respectively, increased by 19.6% (2.01 × 10^3^ Pa/m versus 1.68 × 10^3^ Pa/m) and 22.6% (2.60 × 10^4^ Pa/m versus 2.12 × 10^4^ Pa/m).

One of the main limitations in the current study was that we did not enroll healthy subjects as comparison. Because the aim of this paper was to introduce a medical imaging-based numerical method to assess the instant hemodynamic response during EECP treatment and to conduct a pilot study of the influence of EECP on WSS and its fluctuations in carotid bifurcation with mild plaque.

## 9. Conclusions

We suggest that the framework of patient-specific numerical approach developed in the current paper can be potentially used in clinics for the assessment of instant hemodynamic response in the carotid artery during EECP treatment and in turn may play a role on improvement of the treatment strategies for better clinical outcome. Meanwhile, findings of this paper show that EECP treatment induced a significant augmentation of blood perfusion and WSS level in the carotid artery, which may be the main hemodynamic mechanism underlying its good clinical effect for treatment of the ischemic cerebrovascular diseases and the long-term effect for inhibition of the atherosclerosis lesion.

## Figures and Tables

**Table 1:** Results of specific mesh-independent study.

	Mesh 1	Mesh 2	Mesh 3	Mesh 4
Mesh quantity	259706	419466	720085	907589
AWSS, (Pa) (pre-EECP)	7.772	7.921	7.966	7.991
AWSS, (Pa) (during EECP)	9.043	9.236	9.359	9.372

**Table 2:** Hemodynamic statistics before and during EECP intervention for the whole model and over the cardiac cycle.

	AWSS (Pa)		OSI		AWSSG (Pa/m)	
	Pre-EECP	During EECP	Pre-EECP	During EECP	Pre-EECP	During EECP
Max	38.69	44.90	0.48	0.47	2.12 × 10^4^	2.60 × 10^4^
Min	0.73	0.70	3.2 × 10^−7^	2.1 × 10^−6^	93.11	97.87
Mean	6.76	7.90	0.041	0.042	1.68 × 10^3^	2.01 × 10^3^

## Data Availability

The data used to support the findings of this study are available from the corresponding author upon request.
